# Adaptive behavior in Chinese children with Williams syndrome

**DOI:** 10.1186/1471-2431-14-90

**Published:** 2014-04-04

**Authors:** Chai Ji, Dan Yao, Weijun Chen, Mingyan Li, Zhengyan Zhao

**Affiliations:** 1 Department of child health care, The Children’s Hospital of Zhejiang University School of Medicine, 57# zhugan road, Hangzhou, Zhejiang Province, China

**Keywords:** Williams syndrome, Children, Adaptive behavior

## Abstract

**Background:**

Williams syndrome (WS) is a neurodevelopmental disease characterized by compelling psychological phenotypes. The symptoms span multiple cognitive domains and include a distinctive pattern of social behavior. The goal of this study was to explore adaptive behavior in WS patients in China.

**Methods:**

We conducted a structured interview including the Infants-Junior Middle School Students Social-life Abilities Scale in three participant groups: children with WS (n = 26), normally-developing children matched for mental age (MA, n = 30), and normally-developing children matched for chronological age (CA, n = 40). We compared the mean scores for each domain between the three groups.

**Results:**

Children with WS had more siblings than children in the two control groups. The educational level of the caregivers of WS children was lower than that of the control children. We found no differences in locomotion, work skill, socialization, or self-management between the WS and MA groups. WS children obtained higher scores of self-dependence (*df* = 54, Z = −2.379, *p* = 0.017) and had better communication skills (df = 54, Z = −2.222, *p* = 0.026) compared with MA children. The CA children achieved higher scores than the WS children for all dimensions of adaptive behavior.

**Conclusions:**

WS children have better adaptive behavior skills regarding communication and self-dependence than normal children matched for mental age. Targeted intervention techniques should be designed to promote social development in this population.

## Background

Williams syndrome (WS) is a neurogenetic disorder that occurs in about 1 in every 8,000 live births. WS is caused by the contiguous deletion of 26–28 genes on chromosome 7q11.23. Individuals with WS exhibit compelling psychological phenotypes, including cognitive strengths and weaknesses, and display unique patterns of social behavior [1] An increased appetitive drive toward social interaction is one of the most significant social phenotypes of WS [2]. People with WS may frequently approach others, including strangers, with a disregard for potentially negative consequences. In the process of raising children with WS, many parents and caregivers are challenged by the task of teaching their children to behave in socially acceptable ways.

“Adaptive behavior” refers to the functioning of an individual in his or her social environment. Evaluation of adaptive behavior includes several aspects, such as communication, socialization, daily living, and motor skills. Using some form of the Vineland Adaptive Behavior Scales (VABS) [3], children with WS have been found to have strong socialization (especially interpersonal skills related to initiating social interaction) and communication skills, and poor daily living and motor skills, relative to their overall level of adaptive functioning. Some researchers have reported that the adaptive behaviors of people with WS are poor compared with those of the normal population [4-6].

Unlike Down syndrome, which is the most common cause of inherited intellectual disability, WS is a relatively rare disease that is generally diagnosed only by pediatricians in big cities. In China, individuals with WS are considered to have a general intellectual disability. Although they face a variety of life-long challenges, these individuals may marry and have children, although their offspring may also have genetic abnormalities.

The goal of this study was to explore social ability and behavioral development in children with WS in China. We chose a systematic approach in which children with WS were compared with normal children who were matched for both mental age and chronological age. To the best of our knowledge, no studies have reported on social ability and behavioral development in Chinese children with WS. The aim of this study was to determine whether an etiology-specific profile exists for social adaptation in Chinese people with WS. To this end, we used the Infants-Junior Middle School Students’ Social-Life Abilities Scale [7].

## Methods

### Participants

A total of 96 individuals participated in the study, including 26 children with WS, 30 children matched for mental age (MA group) and 40 children matched for chronological age (CA group). The children with WS were recruited at the Children’s Health Care outpatient clinic of the Children’s Hospital (Zhejiang University, School of Medicine) between May 2010 and November 2012. They were diagnosed clinically and had a confirmed deletion of the elastic gene on chromosome 7q11.23, determined using fluorescence in situ hybridization (FISH) (Vysis Inc., Downers Grove, IL, USA). The control participants were recruited from kindergarten groups and primary schools. All the parents or guardians of the control participants reported their children to be in good physical and mental health, and stated no known previous or current neurological or psychiatric diseases or current drug intake among the control children. The control participants were the same as those in “Study on the Social Adaptation of Chinese Children with Down Syndrome”, a recent study completed by one of the authors of this study [8]. The two research projects were initiated simultaneously and had planned to share the participants in the control groups. The prevalence of Down Syndrome is substantially higher than that of WS, so the study focused on Down Syndrome reached completion earlier than this study.

The mean age of the participants was 89.66 + 36.66 months (ranging from 36–159 months) for the WS patients, 36.17 + 10.65 months (ranging from 19–65 months) in the MA group and 92.13 + 30.83 months (ranging from 43–144 months) in the CA group. All of the children lived at home, although 5 of the WS children (19.2%) were not living with their biological parents. 7 of the study participants (4 WS children, two children in the CA group, and one in the MA group) had one sibling. There were no significant differences in the child’s sex, family income, or parental marital status (Table 1).

**Table 1:** Sociodemographic profile of the sample population

**Characteristics**	**WS group (n = 26)**	**CA group (n = 40)**				**MA group (n = 30)**			
		**Mean + SD/n**	**t/x2**	**df**	**p value**	**Mean ± SD/n**	**t/x**^**2**^	**df**	***p *** **value**
Age	89.66 ± 36.66	92.13 ± 30.83	t = −0.294	64	0.770	36.17 ± 10.65	t = 7.638	54	0.000
Sex			x^2^ = 0.000	1	1.000		x^2^ = 0.249	1	0.618
Male	13	20				17			
Female	13	20				13			
PPVT raw score	31.96 ± 24.71	101.42 + 24.55	t = −11.205	64	0.000	28.3 + 13.81	t = 0.696	54	0.489
Family characteristics			x^2^ = 4.047	1	0.044		x^2^ = 1.649	1	0.199
Nuclear family, only child	14	32				13			
Nuclear family, Multiple children	4	2				1			
Extended family	8	6				16			
Family income (yuan/month)			x^2^ = 0.003	1	0.954		x^2^ = 0.267	1	0.649
>7500	13	20				16			
3000-7500	11	14				11			
<3000	2	6				3			
Caregiver Education (yrs)			x^2^ = 4.097	1	0.043		x^2^ = 7.045	1	0.008
>12	9	23				18			
9–12	9	7				8			
<9	8	10				4			

Written informed consent was obtained from the legal representative (relatives and/or guardians) of each study participant.

### Procedure

Trained physicians conducted structured interviews with each child and his/her parents or guardians in a quiet room at the outpatient department of the children’s hospital at Zhejiang University. Verbal mental age was tested using the Peabody Picture Vocabulary Test (PPVT) [9]. We employed the PPVT in this study as it has been used in previous studies on children with developmental disorders, and seems more suitable than the WISC-R for testing children with intellectual disabilities [8]. Despite this choice, it was not possible to calculate individual IQ scores and mental ages for most of the WS patients because their chronological ages exceeded the age range of the test (i.e. 3 years 6 months to 8 years 6 months). Therefore, we adopted the raw PPVT score as the measurement of verbal mental age for the purposes of our study.

For the PPVT, each participant was seated in front of a computer with a touch screen. The participant chose pictures on the screen by pointing with his/her finger, and the computer automatically recorded the results. We then used the Infants-Junior Middle School Students’ Social-Life Abilities Scale to evaluate adaptive behavior in each child. The parents or guardians were interviewed about the performance of their children. We also obtained information about a variety of sociodemographic variables, including sex, age, medical history, family structure, family income, caregivers’ education, and newborn history.

### Measures

#### Adaptive behavior

We used the Infants-Junior Middle School Students’ Social-Life Abilities Scales to assess six dimensions: self-dependence, locomotion, work skills, communication, socialization, and self-management. The interviewer asked the parents or guardians to identify the skill level that their child had exhibited in the past 6 months. This scale is a 132-item questionnaire measuring an individual’s personal and social competence from six months to 14 years old. A perfect score is 132 (31 for self-dependence, 18 for locomotion, 20 for work skills, 23 for communication, 22 for socialization, and 18 for self-management). Self-dependence included skills such as drinking, getting dressed, and bathing. Locomotion included sitting, running, and going to school. Work skills included drawing, opening a bottle, and cooking. Communication included speaking, reading, and writing. Socialization included showing an interest in others, having a preferred friend, and engaging in school activities. Self-management included doing something by oneself, taking care of elderly and younger people, saving money, and planning.

We used the Chinese Revised Version of the PPVT to assess cognitive development with verbal mental age matching. There were 120 trials in total, with four pictures to choose from in each trial. In the trials, each child was asked to select the picture from the four that most closely resembled what the interviewer had said. For example, when the interviewer said “girl” the child would be expected to choose the picture showing a girl. The maximum score was 120 points, and the raw score was the total number of correct choices.

#### Data analysis

The children were divided into three groups for analysis (WS, MA, and CA groups). All information obtained during the questionnaire was entered into an SPSS database and analyzed using the SPSS 13.0 software package for Windows. Statistical tests were performed as two-sided tests. A p-value of 0.05 or less indicated statistical significance. T-tests were used to compare two continuously distributed variables, and a non-parametric test (Mann–Whitney U-test) was used for variables with distributions other than normal, i.e. for all factors of adaptive behavior. Chi-squared tests were based on one or more degrees of freedom, as indicated in Table 1.

## Results

### Sociodemographic profile of the participants

The sociodemographic characteristics and newborn history are shown in Table 1. Following the matching procedure, we found no significant differences between the participants in the MA and WS groups in terms of raw PPVT score, sex, family income, or family characteristics. Similarly, we found no differences in age, sex, or family income between the WS and the CA groups.

WS families had a different family structure compared with families from the CA group: 15 percent of WS families had more than one child (CA, 5%; MA, 3.3%). Additionally, the educational level of caregivers with WS children was lower than that of the controls.

We were able to measure the IQs of 19 WS children using the WISC-R. The highest score achieved was 61, and 4 participants had IQ scores below 45 (21%). 17 WS children (65.4%) attended kindergarten or elementary school, with only 3 of these children receiving special educational interventions at school, and the other children stayed at home.

#### Adaptive behavior

As mentioned above, a perfect score on the Infants-Junior Middle School Students’ Social-Life Abilities Scale is 132 points, with each item worth one point. The score for each dimension is the total number of items in that dimension that each participant passed. We found no differences between the WS group and the MA group in terms of locomotion (Z = −1.389, *p* = 0.165), work skill (Z = −1.621, *p* = 0.105), socialization (Z = −0.621, *p* = 0.535), and self-management (Z = −1.955, *p* = 0.051). WS children scored higher than MA children in terms of self-dependence (Z = −2.379, *p* = 0.017) and communication skills (Z = −2.222, *p* = 0.026) (Table 2). Children in the CA group achieved higher scores than WS children for all dimensions of adaptive behavior (Table 3).

**Table 2:** Adaptive behavior in the WS and CA groups

**Task**	**WS Group**		**CA Group**		**z**	***p *** **value**
	**Mean ± SD**	**Mean rank**	**Mean ± SD**	**Mean rank**		
Self-dependence	17.38 ± 7.21	19.04	26.45 ± 4.86	42.9	−4.946	<0.0001
Locomotion	7.58 ± 3.65	21.87	11.35 ± 3.29	41.06	−3.992	<0.0001
Work skills	7.85 ± 3.80	18.38	14.40 ± 3.88	43.32	−5.171	<0.0001
Communication	9.73 ± 4.27	17.62	18.32 ± 4.81	43.82	−5.449	<0.0001
Socialization	8.85 ± 3.39	16.67	17.13 ± 4.24	44.44	−5.757	<0.0001
Self-management	5.88 ± 2.90	18.08	13.78 ± 4.63	43.52	−5.238	<0.0001

Data are shown as mean ± standard deviation and mean rank.

The p values were calculated using the Mann–Whitney U test.

WS, Williams syndrome; CA, chronological age.

**Table 3:** Adaptive behavior in the WS Group and MA groups

**Task**	**WS Group**		**MA Group**		**z**	***p *** **value**
	**Mean ± SD**	**Mean rank**	**Mean ± SD**	**Mean rank**		
Self-dependence	17.38 ± 7.21	34.06	13.27 ± 4.68	23.68	−2.379	0.017
Locomotion	7.58 ± 3.65	31.71	6.37 ± 1.56	25.72	−1.389	0.165
Work skills	7.85 ± 3.80	32.27	6.17 ± 1.98	25.23	−1.621	0.105
Communication	9.73 ± 4.27	33.67	7.57 ± 2.49	24.02	−2.222	0.026
Socialization	8.85 ± 3.39	29.94	8.50 ± 2.16	27.25	−0.621	0.535
Self-management	5.88 ± 2.90	33.04	4.60 ± 1.99	24.57	−1.955	0.051

Data are shown as mean ± standard deviation and mean rank.

The p values were calculated using the Mann–Whitney U test.

WS, Williams syndrome; MA, mental age.

## Discussion

The purpose of this study was to obtain more information about the adaptive behavior of children with WS in China. As no thoroughly validated instruments for measuring cognitive development are available in Chinese, we used the PPVT to measure intelligence level.

In Western countries, several studies using a variety of standardized measures (e.g., Wechsler Intelligence Scales, Kaufman Brief Intelligence Test–K-BIT, Stanford Intelligence Scales and Differential Ability Scales–DAS) have reported a high incidence of intellectual disability in people with WS, ranging from mild to moderate [10]. Previous studies have found that individuals with WS score between 55 and 69 on intelligence assessments, while the standard global score for people with intellectual disabilities is between 40 and 90 [11-14]. These scores appear to remain stable during adulthood [12,13,15].

Many different strategies can be effective in treating people with disabilities in developed countries. Children with WS in developed countries may have greater access to appropriate interventions, and their parents may be more educated about treatment options. However, most of the diagnoses of children with WS in China have been made recently compared with developed countries. The highest IQ score that we found in our sample population was 61, and only 3 of the children with WS that we studied had received special interventions. The low level of cognition and social competence that we observed suggests that Chinese families with children with WS lack appropriate professional help, which is necessary to promote the ability of their children early on in development.

Family structure plays an important role in the cognitive and social development of children. Since the implementation of the family planning policy in China, family characteristics have undergone great changes. Many couples who are permitted to have a second child choose to have only one child, although those who have disabled children may often choose to have two children [16]. The nuclear family with one child has become the norm in Chinese society. When a family has a new baby or toddler, or has a handicapped child, the grandparents are usually invited to live together with the young family, for economic reasons or as a matter of custom. As the child grows up, the grandparents generally move out to live by themselves, often while the child is relatively young. Grandparents of children with WS often continue to live with the family to provide additional support. Therefore, the nuclear family structure with one child is the standard social living environment for healthy children the same age as those in the CA group in our study, although this is not the case for children like those in our WS or the MA groups. A handicapped child is usually treated as the most important member of the family. However, the practice of spoiling disabled children may slow the development of their abilities, because these children may have fewer chances to practice skills related to independence.

In addition to family structure, the educational level of the caregivers (mainly parents) plays an important role in the development of social adaptation [17]. Compared with typically-developing children, we found the educational level of parents with children with WS to be significantly lower. In more prosperous regions of China, prenatal screening has become a popular option for well-off parents. To some degree, parents with a lower educational level are less likely to participate in routine examinations during pregnancy, increasing their chance of missing prenatal diagnoses of many genetic diseases. After the birth of a child with WS, well-educated parents may provide a better environment for the growth and development of their disabled child and may participate in early intervention programs more readily. Early intervention can help many children with WS to develop their full cognitive and social potential and live productive lives well into adulthood.

In our study, children in the CA group received higher scores than those in the WS group for all dimensions of adaptive behavior. This is consistent with the opinion that the development of cognition is associated with the development of adaptive behavior and improvement in quality of life [18,19]. The development of adaptive behavior is influenced by language, motor development and executive function. A focus on cognitive skills appears to improve individual potential and social adaptation, while traditional types of education and training are considered to be less helpful for children with developmental disabilities.

We found no differences between children with WS and those in the MA groups in terms of locomotion, work skill, socialization, and self-management, while children with WS obtained higher scores of self-dependence and communication. This result is consistent with the notion that intellectual disability does not inhibit learning and adaptation. Greer examined the performance of children with WS aged 4–18 years and found that standard scores on the Socialization and Communication scales were significantly higher than standard scores on the Daily Living Skills or Motor Skills scales [6]. On another parent-interview measure, the Scales of Independent Behavior - Revised, children with WS performed significantly better on the Social Interaction and Communication Skills scale compared with the remaining scales [20]. In this study, we found that, with the exception of the communication domain, children with WS had higher levels of self-dependence than children in the MA group. This result has not been reported previously. Generally, the mental age of the children with WS in our study was about 3 years old. Items related to self-dependence for this age group include finishing a meal independently and getting dressed (or undressed), which are not necessarily skills that are taught to Chinese children at this age. Basic living skills in people with WS significantly improve after many years of training, although they will generally be much lower than those of people in a CA-matched group.

Menghini found that individuals with WS had difficulties with movement, visuospatial construction, and executive functions, which contribute to the difficulty level of daily skills, such as dressing, cleaning, or playing games [21]. Indeed, locomotion, work skills, and self-management are dependent on motor skills. Children with WS may develop verbal and auditory rote memory abilities, and may develop strong personality characteristics that aid in social adaption, such that they are comparable to the children in the MA-matched group in our study. It is possible that overprotection from caregivers reduces the opportunities for children with WS to develop their skills; especially as family members learn to communicate with and satisfy the child. This may prevent the development of social adaption strategies.

## Conclusion

Children with WS have better adaptive behavior skills in some domains than would be expected given their IQ scores. They require special health care and early interventions to ensure optimal development, which has a significant impact on their families. Independence training for the affected individuals and education for their parents regarding effective interventions might reduce dependency in adulthood and promote heightened skills and abilities. The findings of this study may be useful in the design and implementation of targeted intervention techniques to promote healthy social development in affected individuals. Further investigations are needed to address the factors involved in delayed development among Chinese children with WS.

### Ethics

The protocols and procedures of this study were approved by the ethics committee of the Children’s Hospital at Zhejiang University [2009031]. We obtained signed informed consent from the parents of each participant.

## Competing interests

The authors received financial support from Zhejiang Province innovation team for early screening and intervention of birth defects (Grant No. 2010R50045). The authors declare no conflict of interest in relation to academic, religious or political aspects.

## Authors’ contributions

CJ and ZZ designed the study and wrote the manuscript. WC performed the statistical analysis. Dan Yao and ML supervised the analysis and interpretation of results and contributed to the revision of the manuscript. All of the authors agreed on the final version of the manuscript.

## Authors’ information

Chai Ji is an associate chief pediatrician. She has been working in child healthcare for more than ten years. Her specialty is developmental and behavioral pediatrics, and she focuses on inherited intellectual disabilities and chromosomal genetic diseases. Zhengyan Zhao is a Qiushi Professor at Zhejiang University and a Mid-Aged Expert with Special Contributions at the Ministry of Health. Prof. Zhao is a doctoral advisor and chief physician. His specialty is developmental and behavioral pediatrics. Mingyan Li, PhD, is a pediatrician who works as a resident in the department of child health care. She mainly focuses on the diagnosis and treatment of nutritional diseases, and the role of follow-up appointments on the early growth and development of high-risk infants. Dan Yao and Weijun Chen are both pediatricians with medical master’s degrees. They work as residents in the department of child health care. Dan Yao’s specialty is developmental and behavioral pediatrics. Weijun Chen’s specialty is the diagnosis and treatment of neurodevelopmental disease.

## Pre-publication history

The pre-publication history for this paper can be accessed here:

http://www.biomedcentral.com/1471-2431/14/90/prepub
